# *Plasmodium falciparum *spermidine synthase inhibition results in unique perturbation-specific effects observed on transcript, protein and metabolite levels

**DOI:** 10.1186/1471-2164-11-235

**Published:** 2010-04-12

**Authors:** John VW Becker, Linda Mtwisha, Bridget G Crampton, Stoyan Stoychev, Anna C van Brummelen, Shaun Reeksting, Abraham I Louw, Lyn-Marie Birkholtz, Dalu T Mancama

**Affiliations:** 1 CSIR Biosciences, PO Box 395, Pretoria, 0001, South Africa; 2 Department of Biochemistry, University of Pretoria, Pretoria, Gauteng, 0002, South Africa

## Abstract

**Background:**

*Plasmodium falciparum*, the causative agent of severe human malaria, has evolved to become resistant to previously successful antimalarial chemotherapies, most notably chloroquine and the antifolates. The prevalence of resistant strains has necessitated the discovery and development of new chemical entities with novel modes-of-action. Although much effort has been invested in the creation of analogues based on existing drugs and the screening of chemical and natural compound libraries, a crucial shortcoming in current Plasmodial drug discovery efforts remains the lack of an extensive set of novel, validated drug targets. A requirement of these targets (or the pathways in which they function) is that they prove essential for parasite survival. The polyamine biosynthetic pathway, responsible for the metabolism of highly abundant amines crucial for parasite growth, proliferation and differentiation, is currently under investigation as an antimalarial target. Chemotherapeutic strategies targeting this pathway have been successfully utilized for the treatment of Trypanosomes causing West African sleeping sickness. In order to further evaluate polyamine depletion as possible antimalarial intervention, the consequences of inhibiting *P. falciparum *spermidine synthase (PfSpdSyn) were examined on a morphological, transcriptomic, proteomic and metabolic level.

**Results:**

Morphological analysis of *P. falciparum *3D7 following application of the PfSpdSyn inhibitor cyclohexylamine confirmed that parasite development was completely arrested at the early trophozoite stage. This is in contrast to untreated parasites which progressed to late trophozoites at comparable time points. Global gene expression analyses confirmed a transcriptional arrest in the parasite. Several of the differentially expressed genes mapped to the polyamine biosynthetic and associated metabolic pathways. Differential expression of corresponding parasite proteins involved in polyamine biosynthesis was also observed. Most notably, uridine phosphorylase, adenosine deaminase, lysine decarboxylase (LDC) and S-adenosylmethionine synthetase were differentially expressed at the transcript and/or protein level. Several genes in associated metabolic pathways (purine metabolism and various methyltransferases) were also affected. The specific nature of the perturbation was additionally reflected by changes in polyamine metabolite levels.

**Conclusions:**

This study details the malaria parasite's response to PfSpdSyn inhibition on the transcriptomic, proteomic and metabolic levels. The results corroborate and significantly expand previous functional genomics studies relating to polyamine depletion in this parasite. Moreover, they confirm the role of transcriptional regulation in *P. falciparum*, particularly in this pathway. The findings promote this essential pathway as a target for antimalarial chemotherapeutic intervention strategies.

## Background

At present, antimalarial drug resistance is a critical threat and the need for compounds with novel modes-of-action is imperative. Malaria pathogenesis is exhibited during the asexual erythrocytic cycle of *Plasmodium falciparum *in the human host and a variety of parasite processes and diverse targets are potentially available to inhibit parasite proliferation. One of these targets is the biosynthesis of polyamines - essential and ubiquitous small, aliphatic compounds containing two or more amino groups, which in eukaryotes mainly include putrescine, spermidine and spermine [1]. A fourth polyamine, cadaverine, is a structural analogue of putrescine with functions similar to the other polyamines though better characterized in prokaryotes [2]. At physiological pH, these polycations interact electrostatically with various anionic macromolecules such as DNA, RNA, ATP, phospholipids and proteins [1,3]. These interactions can alter DNA conformation, regulate replication and transcription, strengthen membranes, regulate ion channels and protect DNA and phospholipids from oxidative stress [1,3-6].

Ornithine decarboxylase (ODC) and S-adenosylmethionine decarboxylase (AdoMetDC) usually regulate polyamine metabolism and inhibitors against these enzymes are being applied in diverse therapies ranging from tumour suppressors to the treatment of West African sleeping sickness (*Trypanosoma brucei gambiense*), validating polyamine metabolism as a target for drug intervention in these protozoan parasites [1]. In *P. falciparum *AdoMetDC and ODC are encoded by a single polypeptide to form a unique bifunctional protein (PfAdoMetDC/ODC) [7]. This enzyme has been the main focus of studies assessing polyamine metabolism as a drug target in the parasite. However, traditional inhibitors of the polyamine pathway aimed at these proteins have cytostatic effects with curative rates only achieved in combination with polyamine analogues in murine malaria models [8]. A previous study focused on PfAdoMetDC/ODC indicated that polyamine depletion resulted in transcriptional arrest [9], which manifested as a halt in the parasite's intraerythrocytic developmental cycle (IDC). Therefore, polyamines appear to be essential molecules for parasite survival and promising targets for antimalarial therapeutic intervention [10].

Spermidine is synthesized from putrescine and decarboxylated S-adenosylmethionine (dcAdoMet) through the aminopropyltransferase action of spermidine synthase (SpdSyn) [11]. In *P. falciparum*, this protein has the additional and unique function of being responsible for the low level production of spermine [12,13]. The relative paucity of polyamine studies focused on PfSpdSyn may belie the importance and essential nature of this enzyme, reflected by the need for spermidine in the synthesis of hypusine, eukaryotic initiation factor 5A and its involvement in *P. falciparum *DNA polymerase and topoisomerase I and II [14]. In addition, the lack of polyamine interconversion in *P. falciparum *implicates the flux through PfSpdSyn as the determinant of spermidine levels [10,15]. In support of the latter, inhibition of PfSpdSyn activity with either substrate or transition state analogues has been shown to totally block *P. falciparum *schizogony due to depletion of spermidine [16]. This contrasts with other cell lines in which growth rates were only moderately affected, due to the maintenance of cellular polyamine levels by the interconversion pathway [17]. Evidence for the rescue of PfSpdSyn inhibition by exogenous polyamines is contradictory and seems to be dependent on the inhibitor used: reversibility was found with the putrescine analogue dicyclohexylamine [16] but not with the more potent inhibitors, *trans-*4-methylcyclohexylamine (4 MCHA) or 5-amino-1-pentene (APE) [12].

The modes of gene regulation in *P. falciparum *is currently controversial, with evidence supporting the dominant role of post-transcriptional control on the one hand [18-21] and evidence mounting for the presence of transcriptional control, particularly in response to external perturbations on the parasite, on the other [9,22-27]. A recent functional genomics study of co-inhibited PfAdoMetDC/ODC demonstrated that perturbation-specific compensatory transcriptional responses are induced within the parasite to alleviate the effects of polyamine depletion [9], similar to a previous study of ODC-inhibited malaria parasites [28]. In the investigation presented here, these two studies were extended to analyze the effect of inhibition of PfSpdSyn. Remarkably, unique inhibitor-specific effects were detected on several levels in this study. Moreover, perturbation-specific effects relating to polyamine depletion, as reported in the previous studies, were confirmed, thus corroborating the role of transcriptional regulation in polyamine metabolism of *P. falciparum*.

## Results

### Morphological Study

A number of compounds have been shown to inhibit spermidine synthase activity in *P. falciparum *[12,16]. Some of these drugs include dicyclohexylamine, agmatine, 1,7-diaminoheptane, 4 MCHA, APE, (*S, R*)-dcAdoMet, 1-aminoxy-3-aminopropane (APA) and cyclohexylamine. Of these, only dicyclohexylamine and cyclohexylamine are commercially available, with cyclohexylamine being more potent and inhibiting *P. falciparum *growth with an IC_50 _of 198 μM compared to 342 μM, for dicyclohexylamine [12]. The potency of cyclohexylamine was also reflected in its inhibition of recombinantly expressed PfSpdSyn (IC_50 _of 19.7 μM, [12]). This motivated the use of cyclohexylamine to obtain PfSpdSyn inhibition in *in vitro P. falciparum *cultures and investigate the resultant effects on parasite morphology, transcriptome, proteome and selected polyamine metabolites.

**Figure 1:**
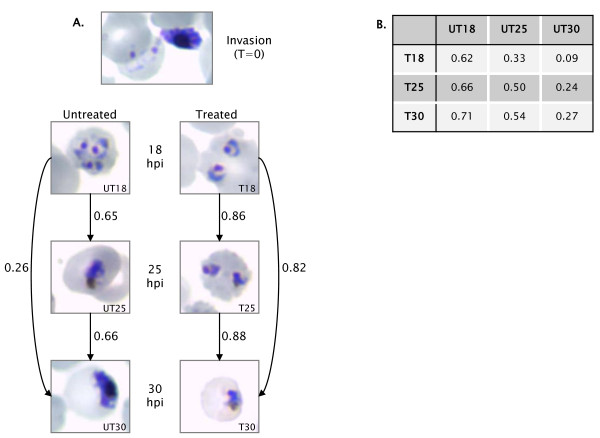
**Morphological analysis of *Plasmodium falciparum *following inhibition of PfSpdSyn with cyclohexylamine**. (A) Parasites treated with cyclohexylamine at invasion were arrested in the early trophozoite stage, correlating close to the time of maximal expression of this transcript in the intaerythrocytic developmental cycle (IDC). Untreated parasites matured through mid- into late trophozoites at comparable time points. Arrows indicate the Pearson correlation (r) between samples. In its 48 h lifecycle, the expression of PfSpdSyn is maximally expressed at 18 hours post-invasion (hpi) in a window of expression from 10-40 hpi. (B) Pearson correlations of untreated and treated samples, showing poor correlations of untreated samples to their treated counterparts at later time points (25 and 30 hpi). The approximate time of arrest (between 10 and 18 hpi) is indicated by the highest correlations to the untreated samples at 18 hpi.

A morphological study was performed on PfSpdSyn inhibited parasites to ensure complete growth arrest at the drug concentration used (2 mM cyclohexylamine) and to determine sampling times for subsequent transcriptome, proteome and metabolite analyses. Drug treatment was initiated during parasite invasion. Treated parasites were arrested in the early trophozoite stage, while untreated parasites had matured from early to mid/late trophozoites at 30 hours post invasion (hpi) (Figure 1A). There were no morphologically detectable differences between treated and untreated parasites in the earlier stages (ring stage parasites, results not shown), limiting the possibility of off-target effects of the drug treatment prior to PfSpdSyn inhibition. These results correlate with findings indicating a stage-specific expression of PfSpdSyn with maximum accumulation of transcript and protein in trophozoites at 18 hpi [12] (ranging from 10 - 40 hpi, Malaria IDC Strain Comparison Database [29]), and a morphological visible difference in parasites treated with cyclohexylamine only after PfSpdSyn is produced (Figure 1A). Based on these results, sampling times for subsequent transcriptome and proteome analyses were chosen at early (18 hpi), mid (25 hpi) and late (30 hpi) trophozoite stages, thereby spanning the period in which PfSpdSyn is maximally expressed and during which parasite growth arrest is observed. Metabolite analyses were performed at 25 hpi.

### Transcriptional profiling of cyclohexylamine-treated *P. falciparum*

Transcriptome analyses of cyclohexylamine-treated compared to untreated parasites employed a reference design [30] for hybridization to an Operon oligonucleotide array, interrogating 7797 70-mer oligonucleotides which represent 4585 unique genes. RNA was extracted from several untreated, unsynchronized *P. falciparum *3D7 cultures and cDNA synthesized for construction of a universal reference RNA (URR) pool resulting in 86% coverage, according to the method of Novoradovskaya (2004) [31]. Technical and biological variation between hybridisations was estimated by Pearson correlations between the individual slides. 5506 datapoints (spots) passing the normalisation filters across all arrays (18 datasets) were utilised for this purpose. Technical variation was calculated as 0.90 ± 0.04 across all replicates. Biological variation was calculated as 0.85 ± 0.10 across all biological replicates. All technical and biological correlations are given in full in Additional file 1: Table S1. Pearson correlations (r) of the relative expression values of the untreated (UT_18_, UT_25 _or UT_30_) and treated (T_18_, T_25 _or T_30_) samples compared to the reference pool utilised 6823 spots (six collated datasets) in the analyses. The correlations indicated transcriptional arrest in the parasite following cyclohexylamine treatment (Figure 1B). This is evident from Pearson correlations between treated samples, where T_18 _vs T_25 _= 0.86, T_18 _vs T_30 _= 0.82 and T_25 _vs T_30 _= 0.88, indicating that after cyclohexylamine treatment, parasites were in comparable transcriptional states (Figure 1A). In contrast, in untreated samples, where no arrest was observed at a transcriptional or morphological level, correlations between time points were significantly lower (e.g. UT_18 _vs UT_30 _= 0.26, Figure 1B). Parasite arrest was estimated close to 18 hpi, as can be gauged from correlations between untreated and treated samples (UT_18 _vs T_18 _= 0.62; UT_18 _vs T_25 _= 0.66 and UT_18 _vs T_30 _= 0.71), which indicate the approximate time of transcriptional arrest (close to but not exactly at 18 hpi) leading to the observed cytostasis (Figure 1B). Additionally, this cytostasis correlates well with the expression time of PfSpdSyn spanning 10-40 hpi and reaching maximal expression at 18 hpi (Malaria IDC Strain Comparison Database [29]). The comparison of the cytostatically arrested treated parasites to untreated parasites at 18 hpi (UT18) is therefore essential to identify differences in the transcriptomes due to drug treatment. Parallel time point comparisons indicated lower correlation values (UT_30 _to T_30 _at 0.27) indicating the presence of cell cycle differences between these populations and negating the use of direct time point comparisons. These results are in strong agreement with those obtained by van Brummelen (2009) [9], where the combination of two cytostatic drugs that target the polyamine pathway also resulted in a global transcriptional arrest after polyamine depletion. Subsequently, for analysis of significantly affected transcripts, all samples were compared to UT_18_, thereby following a reference point strategy similar to previous reports [9,26], such that drug-specific events were observed and not only cell cycle/stage differences.

Following analysis of differentially expressed transcripts with the LIMMA package in R, it emerged that a total of 706 unique genes (Additional file 2: Table S2) were differentially regulated (across all time points) at least two-fold when applying a false discovery rate cutoff of 5% (significance of p = 0.05). This amounted to 26.4% of the total unique PlasmoDB identities passing the data normalization and quality filter (2673 in total) on the array and included 387 downregulated and 319 upregulated transcripts. The largest number of transcripts differentially expressed occurred at T_18 _(502 in total), declining to 472 at T_25 _and 221 at T_30 _(Additional file 2: Table S2). Several of these transcripts were differentially expressed at multiple time points.

The transcriptome data was validated by examining relative expression of five transcripts functioning in the polyamine/methionine biosynthetic pathway with quantitative real-time PCR (RT-qPCR) analyses. Primers were designed to specifically amplify adenosine deaminase (PF10_0289), LDC (PFD0285c), uridine phosphorylase (PFE0660c), phosphoethanolamine N-methyltransferase (MAL13P1.214) and spermidine synthase (PF11_0301). Eukaryotic translation initiation factor 3 subunit 10 (PFL0625c) was used as endogenous control, since its transcript was not affected by the drug perturbation throughout the time period analyzed, as deduced from array data (at p < 0.05) and the Malaria IDC Strain Comparison Database [29]. RT-qPCR data was in strong agreement with that obtained from array analyses (Table 1), as indicated by a Pearson correlation value of 0.88 for the two datasets. RT-qPCR analyses confirmed the direction of differential regulation (up or down) as well as where transcripts were deemed to be unchanged by the perturbation (e.g. spermidine synthase). Downregulated transcripts showed good concordance between oligonucleotide array and RT-qPCR analyses (e.g. fold change of adenosine deaminase at T_30 _-2.778 and -3.413, for oligo and RT-qPCR analysis, respectively). Similarly, upregulated transcripts showed good concordance, albeit with a smaller fold-change in RT-qPCR analyses (e.g. LDC, Table 1).

**Table 1:** Correlation of transcripts and proteins of the polyamine biosynthetic pathway following PfSpdSyn inhibition.

	Fold change (relative to control)					
	
Annotation PlasmoDB ID	Hours post invasion	Oligos representing gene	Oligonucleotide array		RT-qPCR	Proteomics
	18	1	-2.304		-3.472	nd
	
Adenosine deaminase, putative PF10_0289	25	1	-2.907		-5.495	nd
	
	30	1	-2.778		-3.413	-3.030

	18	1	-3.559		-4.651	nd
	
Purine nucleoside phosphorylase/uridine phosphorylase, putative PFE0660c	25	1	-3.731		-5.882	-2.222
	
	30	1	-3.367		-4.348	nd

	18	1	-1.961		-3.115	nd
	
Phosphoethanolamine N-methyltransferase, putative MAL13P1.214	25	1	-3.401		-5.495	nd
	
	30	1	-3.774		-4.049	nd

	18	2	-1.157	1.123	-1.185	nd
	
Spermidine synthase PF11_0301	25	2	-1.038	1.300	-1.289	nd
	
	30	2	-1.035	1.320	+1.087	nd

	18	1	+1.324		-1.362	nd
	
Lysine decarboxylase, putative PFD0285c	25	1	+2.426		+1.106	nd
	
	30	1	+1.954		+1.591	nd

	18	1	-1.300		nd	-3.030
	
Ornithine aminotransferase, PFF0435w	25	1	+1.044		nd	nd
	
	30	1	+1.182		nd	nd

	18	1	-1.597		nd	-2.857
	
S-adenosyl methionine synthethase, PFI1090	25	1	-1.631		nd	-2.222
	
	30	1	-1.761		nd	nd

	18	1	1.347			nd
			
Eukaryotic translation initiation factor 3 subunit 10, putative (endogenous control) PFL0625c	25	1	1.210		endogenous control	nd
			
	30	1	1.221			nd

The array and quantitative real-time PCR (RT-qPCR) datasets showed a high degree of concordance revealed by a Pearson correlation (r) of 0.88. Fold changes for transcripts and proteins are given at each time point. Differentially affected genes and their cognate proteins generally exhibited the same direction and level of regulation.

### Drug-induced changes to the *P. falciparum *proteome

To further investigate the effect of the cyclohexylamine perturbation on *P. falciparum*, global protein expression analyses was also performed using 2D gel electrophoresis (2D-GE). Similar to the transcriptome analyses, differential analyses were obtained by comparing drug-treated proteome profiles of the parasites at the three time points (18, 25 and 30 hpi) to the protein profile of untreated parasites at 18 hpi. Using PDQuest (Biorad), a total of 167 protein spots were successfully matched with high confidence (CI ≥ 95%) across three replicate gels representing the parasite's entire 18 hpi untreated proteome. Of these, 159, 155, and 149 spots were matched with high confidence to corresponding 2D-GE spots representing the parasite's drug-treated proteome at 18 hpi, 25 hpi, and 30 hpi respectively. Statistical analysis revealed a total of 38 differentially expressed proteins with at least a two-fold change and a statistical significance of p = 0.05 (Student's T-test). The identities of 21 of these were positively established through MALDI-QTOF-MS and MS/MS (nine at 18 hpi, six at 25 hpi and six at 30 hpi) (Additional file 3: Table S3). Four of these were found to be involved in the polyamine/methionine biosynthetic pathway, namely ornithine aminotransferase (PFF0435w), S-adenosylmethionine synthetase (PFI1090w), uridine phosphorylase, putative (PFE0660c) and adenosine deaminase (PF10_0289). It was not possible to establish the identities of 17 of the 38 differentially expressed proteins, due to their low abundance being beyond the detection limits of the mass spectrometer used. However, the successful identification using mass spectrometry of 21 out of the 38 differentially expressed proteins selected for characterization (i.e. a yield of 55%) compared well to previous 2D-GE studies of Plasmodial proteins which displayed an identification rate of approximately 30% [9,32].

Inhibition of SpdSyn in *P. falciparum *resulted in a general downregulation in expressed proteins. Expression of all but one (a putative pyruvate kinase (PFF1300w)) of the 21 differentially affected proteins identified was found to be downregulated. Of these, six displayed a similar direction of differential expression with their corresponding genes, even though the expression levels varied (Additional file 3: Table S3). For 14 downregulated proteins, no significant change in underlying gene expression was observed following treatment, while in the case of the putative pyruvate kinase (PFF1300w), increased accumulation of this protein was detected in contrast to a relative reduction in underlying levels of mRNA. In previous studies, moderately high correlation between mRNA and protein abundance has been shown for *P. falciparum*, varying in degree of correlation according to the parasite's stage-specific development [33]. However, this effect is not observed for all proteins and their encoding genes, and it has been proposed that this discrepancy in part reflects the contribution of more complex regulatory processing involving post-transcriptional mechanisms and/or protein peptide modification [33]. Thus, in the case of the putative pyruvate kinase (PFF1300w) for example, in-depth analysis to determine the modification status of this sequence at the gene and protein level may shed more light on the contrasting changes observed in expression.

### Selected metabolite profiling of cyclohexylamine-treated *P. falciparum*

**Figure 2:**
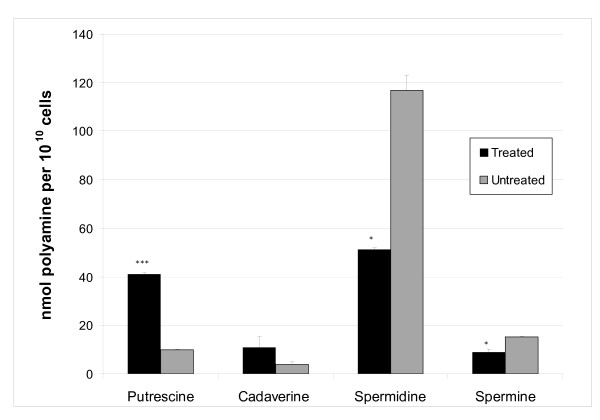
**Levels of selected polyamines following *PfSpdSyn *inhibition**. Values represent the concentration of polyamine per 10^10 ^cells, measured from two independent experiments performed in duplicate. Error bars are given as the standard error of the mean. Statistical significance is indicated by *** equivalent to 99% (p < 0.01) and * to 90% (p < 0.10) determined through a heteroscedastic Student t-test analysis.

Information regarding the effect of PfSpdSyn inhibition on the polyamine profile of *P. falciparum *is still lacking. To investigate polyamine perturbations resulting from treatment of *P. falciparum *with cyclohexylamine, selected metabolite profiling was performed on these parasites. HPLC analyses of polyamines of both cyclohexylamine-treated and untreated *P. falciparum *trophozoites (25 hpi) revealed significant fluctuations in the polyamine content of the parasites (Figure 2). A significant increase (4-fold, p-value 0.004) in the PfSpdSyn substrate putrescine was observed after inhibition. PfSpdSyn inhibition also led to depletion in the levels of the downstream product metabolites, spermidine and spermine (spermidine: ~2- fold decrease (p-value 0.056); spermine: 1.7-fold decrease (p-value 0.099)). Levels of the related polyamine, cadaverine, were assessed to investigate whether a compensatory increase in this metabolite occurs due to induction of LDC transcripts. Levels appeared to be increased but the change was not statistically significant.

### Perturbation responses include polyamine and associated pathways

Differentially affected transcripts and proteins were analyzed in MADIBA [34,35] to ascertain which metabolic pathways were affected by cyclohexylamine challenge. All differentially regulated genes/proteins at each time point were submitted as a cluster. Generally, gene clusters submitted for analyses following clustering are smaller than either of the lists of differentially down- or upregulated genes at each time point (ranging from 81 (upregulated at 30 hpi) to 285 (downregulated at 18 hpi)). Hence, larger p-values were expected from the contingency test (Fisher's exact test). Nonetheless, the polyamine biosynthetic pathway ranked among the pathways most affected (according to lowest p-value) by cyclohexylamine treatment by analyzing the transcriptome data (p-values for polyamine metabolism 0.214, 0.102 and 0.088 for T_18_, T_25_, T_30_, respectively). This effect on the polyamine pathway was confirmed for differentially expressed proteins (0.028, 0.019 and 0.016 for T_18_, T_25 _and T_30_, respectively). The lower p-values for differentially affected pathways elucidated by proteomics analysis were expected since the proteome coverage was not as extensive as the transcriptome analysis (21 proteins vs. 4585 unique genes interrogated) and therefore a smaller amount of proteins were submitted to the contingency test (Additional file 3: Table S3). Comparatively, the glycolytic pathway was scored at 0.322 and 0.416 (T_18 _and T_25, _respectively) and oxidative phosphorylation at 0.106, 0.012 and 0.035 (T_18_, T_25 _and T_30_, respectively) within the transcriptome dataset. For the proteome, the glycolytic score was 0.054 for both T_18 _and T_30, _and not affected at T_25_.

**Figure 3:**
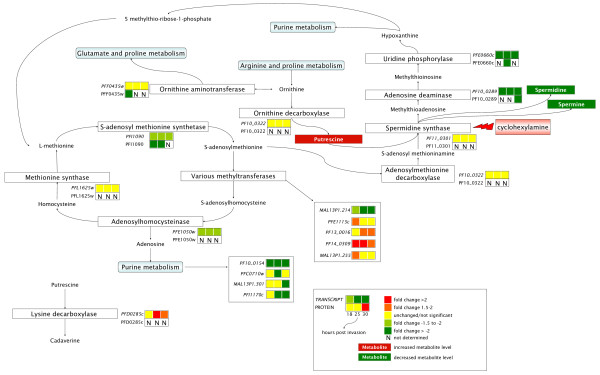
**Regulation of transcripts, proteins and metabolites of the polyamine biosynthetic pathway following cyclohexylamine inhibition**. The relative expression (to untreated control samples) of each transcript and its encoding protein is indicated at each time point sampled. Increased/decreased levels of selected metabolites are indicated. Additionally, regulation of transcripts in purine metabolism and various methyltransferases are indicated.

Differentially affected transcripts and their cognate proteins mapping to the polyamine biosynthetic pathway included adenosine deaminase (maximal fold change -2.907 (transcript) and -3.030 (protein) downregulated) and uridine phosphorylase (fold change -3.731 (transcript) and -2.222 (protein) downregulated) (Table 1, Figure 3). Transcripts encoding LDC (fold change +2.426) were also affected. Furthermore, genes acting in pathways directly linked to the polyamine pathway were differentially regulated. These included four transcripts (ribonucleotide reductase small subunit, putative (PF10_0154), inorganic pyrophosphatase, putative (PFC0710w), protein with aminophospholipid-transporting P-ATPase and guanyl cyclase domains (MAL13P1.301) and thioredoxin reductase (PFI1170c)) in the purine metabolic pathway and five methyltransferases (phosphoethanolamine N-methyltransferase, putative (MAL13P1.214), S-adenosylmethionine-dependent methyltransferase, putative (PFE1115c), methyl transferase-like protein, putative (PF13_0016), protein-L-isoaspartate O-methyltransferase beta-aspartate methyltransferase, putative (PF14_0309) and N6-adenine-specific methylase, putative (MAL13P1.255)) (Figure 3). Additional significantly affected proteins functioning in the polyamine biosynthetic pathway included ornithine aminotransferase (fold change -3.030 in the first time point) and S-adenosylmethionine synthetase (fold change -2.857 in the first time point) (Figure 3, Table 1). Transcripts for the latter were also downregulated but not to the extent of the 2-fold cutoff (Table 1).

Data obtained in this study was compared to that from two previous profiling experiments involving polyamine biosynthetic inhibitors namely DFMO (ODC inhibitor; [28]) and DFMO/MDL73811 (ODC/AdoMetDC inhibitors respectively; [9]). A large degree of overlap was observed for differentially expressed genes and proteins, but effects specific to each treatment were also identified (Table 2). Indeed, 21 upregulated transcripts in the PfSpdSyn inhibition dataset were also observed in the PfAdoMetDC/ODC dataset (12.3% of the latter). Moreover, this number was increased to 60 for overlapping downregulated genes (16% of the PfAdoMetDC/ODC inhibition dataset). Six transcripts of the polyamine pathway were affected in exactly the same manner, whether PfSpdSyn was inhibited with cyclohexylamine (this study) or whether the bifunctional PfAdoMetDC/ODC were co-inhibited with DFMO/MDL73811 (unchanged: spermidine synthase (PF11_0301); upregulated: LDC (PFD0285c); and downregulated: uridine phosphorylase (PFE0660c), adenosine deaminase (PF10_0289), S-adenosylmethionine synthetase (PFI1090w) and adenosylhomocysteinase (PFE1050w) (Table 2)). Conversely, transcripts differentially regulated amongst the three treatments were OAT (PFF0435w, PfSpdSyn inhibition: unchanged (with the exception of protein at 18 hpi), ODC and PfAdoMetDC/ODC inhibition: upregulated), PfAdoMetDC/ODC (PF10_0322, unaffected in PfSpdSyn inhibition, down in PfAdoMetDC/ODC inhibition, not determined in ODC inhibition) and hypoxanthine phosphoribosyltransferase (PF10_0121, unchanged in PfSpdSyn and PfAdoMetDC/ODC inhibition but upregulated by ODC inhibition).

**Table 2:** The regulation of transcripts and proteins of the polyamine pathway in three distinct inhibition experiments.

Annotation	PlasmoDB ID	Cyclohexylamine (PfSpdSyn inhibition) (This work)		DFMO/MDL73811 (PfAdoMetDC/ODC inhibition) [9]		DFMO (ODC inhibition) [28]
		
		Transcript	Protein	Transcript	Protein	Transcript
Ornithine aminotransferase	PFF0435w	**≈**	**↓**	**↑**	**↑**	**↑**

S-adenosylmethionine decarboxylase-ornithine decarboxylase (PfAdoMetDC/ODC)	PF10_0322	**≈**	nd	**↓**	nd	nd

Hypoxanthine phosphoribosyltransferase	PF10_0121	**≈**	nd	**≈**	nd	**↑**

Uridine phosphorylase, putative	PFE0660c	**↓**	**↓**	**↓**	nd	nd

Adenosine deaminase, putative	PF10_0289	**↓**	**↓**	**↓**	nd	nd

Spermidine synthase	PF11_0301	**≈**	nd	**≈**	nd	nd

S-adenosylmethionine synthetase, putative	PFI1090w	↓*	**↓**	**↓**	**↓**	nd

Adenosylhomocysteinase (S-adenosyl-L-homocysteine hydrolase)	PFE1050w	↓*	nd	**↓**	nd	nd

Lysine decarboxylase, putative	PFD0285c	**↑**	nd	**↑**	nd	nd

* = fold change between -1.5 and -2 fold

↓ = downregulated

↑ = upregulated

≈ = unchanged

nd = not determined

A large degree of overlap was observed for the response to polyamine depletion, whether PfSpdSyn, PfAdoMetDC/ODC or ODC alone were inhibited. Also, effects specific to each inhibitor could be gauged.

## Discussion

In this investigation, cyclohexylamine treatment of *P. falciparum *parasites resulted in a perturbation-specific response due to polyamine depletion (regulation of spermidine synthase, LDC, uridine phosphorylase, adenosine deaminase, S-adenosylmethionine synthetase and adenosylhomocysteinase) as well as an inhibitor-specific response due to spermidine synthase inhibition on the transcriptional level (regulation of OAT, PfAdoMetDC/ODC and hypoxanthine phosphoribosyltransferase). These responses will be discussed at the level of the specific polyamine biosynthetic enzymes in more detail.

### LDC

In a previous investigation, inhibition of both decarboxylase activities of the bifunctional PfAdoMetDC/ODC led to the marked upregulation of LDC over a time course [9]. PfSpdSyn inhibition also led to an increase in transcripts encoding LDC, albeit only in one time point in array analyses, and not to the extent of the two-fold cutoff in RT-qPCR analyses. This upregulation of LDC is regarded as a specific compensatory response to polyamine metabolism perturbation resulting in polyamine depletion. Lysine decarboxylation produces cadaverine, a diamine and structural analogue of putrescine, presumably to compensate for the lack of other polyamines as has been shown in plants and partially in *Plasmodium *[36,37].

### Adenosine deaminase and uridine phosphorylase

As expected, downstream enzymes whose function is dependent on the metabolism of polyamines, including adenosine deaminase and uridine phosphorylase, were downregulated upon perturbation of polyamine metabolism on both transcript and protein levels. These were also affected upon PfAdoMetDC/ODC co-inhibition [9]. Therefore, the decreased production of 5-methylthioadenosine, a substrate for adenosine deaminase, after polyamine depletion [14], possibly resulted in a regulatory signal to decrease the production of this protein at the transcriptional level. Consequently, this downregulation may explain the observed decrease in 5-methylinosine following PfAdoMetDC/ODC co-inhibition [14], which manifests in the downregulation of the production of uridine phosphorylase. Ultimately, the downregulation of these proteins would result in the decreased production of precursors for purine metabolism as well as methionine synthesis.

### OAT

During the inhibition of ODC or the co-inhibition of both decarboxylase activities of the bifunctional PfAdoMetDC/ODC [9,28] an upregulation in both the transcript and protein for OAT was observed, potentially to prevent toxic accumulation of ornithine when this metabolite was not used for polyamine production. This was clearly confirmed with a metabolomic analysis which indicated the total depletion of putrescine levels due to the abovementioned inhibition and a homeostatic maintenance of ornithine levels [9,38]. However, upon inhibition of PfSdpSyn, this compensatory increase in OAT transcripts or protein was not observed, which corroborates its specific upregulation when ornithine decarboxylation is prevented.

It would be predicted that inhibition of PfSpdSyn would result in an increase in putrescine concentrations in the parasite and a marked decrease in spermidine concentrations. Metabolite analyses of *P. falciparum *confirmed that inhibition of PfSpdSyn results in a substantial increase in putrescine concentration. Moreover, a decrease was observed in spermidine and spermine concentrations. *P. falciparum *lacks a spermine synthase activity but PfSpdSyn has been shown to uniquely metabolize its product, spermidine, to additionally provide spermine (10% of its activity, [12]). The observation of a decrease in spermine levels after PfSpdSyn inhibition with cyclohexylamine therefore confirms this secondary activity of the protein.

### PfAdoMetDC/ODC

The co-inhibition of the bifunctional PfAdoMetDC/ODC resulted in a downregulation of the transcript for this protein [9]. This effect was not mirrored by PfSpdSyn - the transcript for this protein was not differentially affected and, moreover, putrescine levels increased. Therefore, it seems as if an increase in this metabolite (as was observed for PfSpdSyn inhibition) does not trigger the regulation of PfAdoMetDC/ODC.

### Methionine recycling

Inhibition of PfSpdSyn resulted in a decrease in transcripts for proteins involved in methionine recycling. PfSpdSyn inhibition could presumably also have caused an increase in the decarboxylated form of AdoMet as a product of AdoMetDC. Decarboxylated AdoMet is exclusively used in the synthesis of polyamines [1] and as such, its homeostasis is controlled only by polyamine and methionine metabolism. After co-inhibition of PfAdoMetDC/ODC [14], it was shown that AdoMet levels were homeostatically controlled in the parasite, presumably to prevent a hypermethylated state. The mechanism proposed for the homeostatic control of AdoMet levels in Plasmodia is the downregulation of its synthesis, effectively by downregulating the synthesis of AdoMet synthetase [39]. This downregulation was also observed on both transcript and protein levels in this study after PfSpdSyn inhibition.

Three independent profiling investigations of different polyamine biosynthesis inhibitors indicated highly reproducible perturbation-specific results, as well as specific effects related to the inhibitors used. These findings lend support to the growing body of evidence that implicates a role for transcriptional level control in the response of *P. falciparum *to drug perturbation. The findings further highlight that this organism demonstrates an ability to mount a compensatory response following drug perturbation of the polyamine pathway. For *P. falciparum*, an organism noted for it's adeptness to developing drug resistance, such knowledge is critical to guiding the future direction of drug development strategies seeking to target the polyamine biosynthetic pathway. It is also becoming clear that the phenomenon of directed transcriptional level response to perturbation may occur in select biological processes within the parasite, e.g. in the case of antifolate inhibition such events are not observed [40,41]. This suggests the presence of a highly complex regulatory system which still remains to be fully elucidated, where the dominant regulation may occur at the transcriptional level for some genes, whereas for others it may occur at the post-transcriptional and/or translational level. Recent advances in microarray and mass spectrometry technologies, which now enable simultaneous and in-depth high-throughput analysis of such events, promise to provide greater insight into these phenomena.

## Conclusions

In this investigation, the consequences of inhibiting SpdSyn in *P. falciparum *were evaluated on several levels. Unique polyamine biosynthetic effects, specific to the inhibition of PfSpdSyn, were observed. These effects stem from the specific polyamines perturbed; in this instance the inhibition of the formation of spermidine and spermine. By providing detailed analyses of the consequences of PfSpdSyn inhibition, this work also contributes to efforts aiming to identify drug targets most suited to chemotherapeutic intervention in this pathway.

## Methods

### Culturing and drug treatment

Culturing of parasites and drug treatment experiments were conducted under aseptic conditions. *P. falciparum *isolate 3D7 parasites were cultured in 75 cm^2 ^flasks (Nalgene) in human erythrocytes (blood group A+) and RPMI 1640 medium supplemented with 25 mM HEPES, 0.0088% hypoxanthine, 40 ug/ml gentamycin (all Sigma) and 0.5% Albumax (Invitrogen). Parasites were maintained in continuous culture in a 10 ml volume, and were scaled up to 50 ml for drug treatments. The parasitemia and morphological forms detected in the cultures were determined and estimated by Giemsa (Fluka) stained smears under 1000× light microscopy.

Prior to morphology studies and drug treatment, cultures were synchronized with 5% D-sorbitol [42] for 5 min at 37°C to select for parasites in the ring stage. Sorbitol treatment was repeated two days later. A morphological analysis of the culture was performed in the presence and absence of 2 mM cyclohexylamine (corresponding IC_99 _value) in DMSO over a 48 h period. Smears of the cultures were prepared every 6 h post drug treatment. Fresh cultures were scaled up to 50 ml (5% hematocrit, 10% parasitemia), synchronized twice and treated with 2 mM cyclohexylamine at invasion (K. Lüersen, personal communication). Fifteen milliliters of culture was harvested at late ring/early trophozoite (18 hpi), mid trophozoite (25 hpi) and late trophozoite (30 hpi) stages. Similar volumes were harvested from untreated *P. falciparum *3D7 control cultures at the same time points. Parallel drug treatments were conducted, serving as biological replicates.

### RNA extraction

Erythrocytes from harvested cultures were pelleted, washed once with 1× PBS to remove traces of culture medium, and then frozen at -80°C until RNA extractions were performed. RNA was extracted by the method described by [28] and quantified using the Nanodrop 1000 spectrophotometer (Thermo Scientific). The integrity was confirmed on an Agilent Bioanalyzer.

### Universal Reference RNA pool (URR)

A reference pool of RNA from mixed-stage *in vitro *cultures was constructed. Cultures were grown to high parasitemia, without synchronisation. Twenty-four (24) cultures were harvested and flash-frozen in liquid nitrogen. RNA was extracted as described previously. Care was taken to avoid bias towards any specific life cycle stage. The percentage of spots on the microarray to which the URR hybridized was determined in order to gauge accurate expression ratios for as many spots as possible. Equal amounts of URR were labelled with either Cy3 or Cy5 and hybridized to a microarray (as described in next section). URR coverage was defined as the percentage of spots called "present" when local background-subtracted intensity of a spot exceeded the average background intensity of all spots in that channel [31].

### cDNA synthesis and array hybridization

A reference design was employed for array hybridisation, utilising the URR pool described previously. All solvent-control and drug-treated samples were hybridised to Operon slides, along with the URR. For each time point and each untreated/treated sample, three microarray slides were processed, such that a total of eighteen slides were processed in the study. Two independent cDNA samples (biological replicates) were prepared for each untreated and drug-treated sample at each time point. One of the biological replicate cDNA samples were additionally hybridised to a third slide (representing the technical replicate). cDNA synthesis reactions of 12 μg each were set up for each sample or reference RNA in a total of 50 μl. Prior to denaturation at 70°C for 10 min, 2 μl of oligo d(T) primer (2 μg/μl) and 8 μl of random nanomer primer (0.5 μg/μl) (both New England Biolabs) were added. cDNA synthesis proceeded overnight at 42°C after the addition of an aminoallyl-dNTP mix (Fermentas), DTT, reaction buffer and 200 U of SuperScript III Reverse Transcriptase (Invitrogen). RNA was hydrolyzed by the addition of 10 μl each of 1 M NaOH and 0.5 M EDTA solutions and incubated at 65°C for 10 min. Unincorporated aminoallyl dUTP was removed by purification using the NucleoSpin Extract II Kit (Macherey-Nagel). The eluted cDNA was quantified using a Nanodrop spectrophotometer and similar quantities of test and reference cDNA (typically 2 μg) were dried down to a volume of 2.5 μl in a vacuum drier. Five microliters of a 0.2 M Na_2_CO_3 _buffer, pH 9.0, was added to the cDNA and mixed well. Cy3 (sample) or Cy5 (reference) (2.5 μl) (GE Healthcare) ester was added and the coupling allowed to proceed for 2 h in the dark at room temperature. Uncoupled dyes were removed by purification with the RNeasy Mini Kit (Qiagen). Probe labelling was estimated on a Nanodrop. Similar amounts of labelled probe (typically 100 picomoles) were hybridized to the microarray slides. Slides were prehybridized with a solution containing 5× SSC (from 20× SSC: 3 M NaCl, 1.5 M sodium citrate, pH 7.0), 0.1% SDS and 0.1 mg/mL BSA, prewarmed to 42°C. Prehybridization proceeded in 50 ml Falcon tubes, incubated at 42°C for 45-60 min. Following prehybridization, the slides were washed in three washes of 0.1× SSC before centrifugation for 5 min at 200 × g. The combined Cy-labelled probes (4 μl) were mixed with 36 μl of hybridization buffer (Operon) for a total volume of 40 μl. The probes were denatured at 95°C for 5 min, and subsequently applied to the slide under Lifterslips (Erie Scientific Company). The microarray slides were enclosed in ArrayIt (Telechem International) hybridization chambers and submersed in a heated water bath and incubated for 16-20 h at 42°C. The slides were successively washed in low stringency (2× SSC, 0.5% SDS; heated to 42°C), medium stringency (1× SSC) and high stringency (0.1× SSC) wash buffers for 5 min each. Slides were then centrifuged (5 min, 200 × g). Scanning was performed with an Axon GenePix 4000B scanner (Molecular Devices).

### Microarray data analysis

GenePix results (gpr) files were generated using GenePix 6.0 (Molecular Devices) software, without normalization. For clustering analyses, results files were normalized with DNMAD (Diagnosis and Normalization for MicroArray Data) [43] using print-tip loess. The normalized values were subsequently downloaded and analyzed with the Multiexperiment Viewer (MeV) in the TM4 software suite [44]. Hierarchical Clustering (HCL, average linkage) [45] was performed to estimate technical and biological variation between samples and at which point cytostasis most likely occurred for comparative purposes in downstream analyses. Intensity data for individual slides were imported into LIMMA (linear models for microarray data) in the R computing environment [46]. Pre- and post-normalization diagnostic plots were performed using MARRAY. Data from each microarray slide was normalized using print-tip loess. Data between microarrays was normalized using rquantile normalisation. Pearson correlations were computed in ExCel to estimate variation between technical and biological replicates. Spots excluded from slide correlations and normalisation were those weighted by the limma script or flagged in the Genepix results file (gpr). Additionally, spots termed Alien, Empty, Null and Operon Use Only were excluded from the correlation analyses. These spots were similarly excluded for correlations between untreated and treated samples at each time point following normalisation. Results from biological and slide replicates within each of the time points were collated, and linear models were computed to contrast gene expression between time points. A two-fold change in gene expression was used as cut-off, in conjunction with correction for false discovery (false discovery rate (FDR) = 5%). Normalised data was deposited in the Gene Expression Omnibus (GEO) database, number GSE18075. Analysis of differentially expressed genes was performed in MADIBA ([34] Micro Array Data Interface for Biological Annotation [35]).

### Quantitative Real-Time PCR

cDNA samples prepared for microarray analyses were utilized for quantitative real-time PCR (RT-qPCR) validation of global expression analysis. Briefly, cDNA (with 500 nM of each primer and 1× Power SYBR Green PCR Master Mix (Applied Biosystems)) was amplified in 96 well plates in an Applied Biosystems 7500 Real-Time PCR cycler. Primers were designed using Primer3 Plus [47] with the qPCR module activated. Primer sequences used for polyamine as well as housekeeping genes are available on request. Following enzyme activation at 95°C for 10 min, the cDNA was amplified for 40 cycles (95°C for 15 s, 60°C for 60 s). A melt curve was performed to ascertain primer specificity. Relative expression was calculated using Applied Biosystems Sequence Detection Software.

### Protein extraction and two-dimensional electrophoresis

Parasites from harvested cultures were released from erythrocytes by saponin lysis [48] and the resulting pellet washed five times in ice-cold wash medium (RPMI 1640 medium supplemented with 25 mM HEPES ((4-(2-hydroxyethyl)-1-piperazineethanesulfonic acid), 20 mM sodium bicarbonate and 40 μg/ml gentamycin; all Sigma). A pellet from a 15 ml culture was resuspended in 500 μl lysis buffer (8 M urea, 2 M thiourea, 2% CHAPS (3-[(3-Cholamidopropyl)dimethylammonio]propanesulfonic acid), 65 mM dithiothreitol (DTT) (all Sigma) and 1% IPG buffer pH 3-10 (Amersham)). The sample was freeze-thawed five times in liquid nitrogen and then sonicated on ice (Bandelin Sonopuls sonicator, at power level 65%) using five 10 sec bursts with 10 sec of cooling in between. Care was taken to prevent warming of the sample, which introduces artifacts resulting from urea breakdown and protein carbamylation. The supernatant was clarified by ultracentrifugation at 30 000 × g for 30 min at 4°C [49]. Protein concentration was assayed in duplicate by the RCDC protein assay kit (BioRad) against a standard curve of bovine serum albumin (BSA, BioRad). Isoelectric focusing (IEF) of total protein, 300 μg (analytical gels) and 450 μg (preparative gels), was performed as described by [9]. After IEF, gel strips were equilibrated and cysteine residues reduced and subsequently alkylated by treatment with 1% DTT and 2.5% iodoacetamide (Sigma) in equilibration buffer (50 mM Tris-HCl pH 8.8, 6 M urea, 30% glycerol, 2% SDS and bromophenol blue) for 10 min each prior to second dimension analysis. Two-dimensional electrophoresis (2DE) was subsequently performed on 12.5% polyacrylamide gels at constant current of 40 mA per gel for three hours on the Protean II Cell system (BioRad). After 2DE, analytical gels were stained with SyproRuby fluorescent stain (BioRad) and preparative gels with PageBlue Protein stain (Fermentas) according to the suppliers' protocol. Gels were imaged with Pharos FX imager (BioRad) and differential analysis performed using PDQuest™ (V 8.0.1; BioRad) software on a set of three independent gels per experimental condition. Data comparison was carried out against the untreated protein profile at 18 hpi. Spot intensity was normalized using the "local regression" model to compensate for non-expression-related variations in spot intensity. Images were analyzed for quantitative differences, using two-fold difference in expression with a t-test significance level of 95% applied.

### Protein identification

#### In-gel digestion

Protein spots of interest were manually excised from PageBlue stained gels. Low abundant proteins were excised from more than one gel and gel plugs pooled to increase protein yield for mass spectrometry (MS) analysis. The number of gels that spots were excised from depended on the intensity of the protein spot. Protein spots were subjected to in-gel trypsin digestion and the resulting peptide mixture was purified using Stage tips^® ^(Proxeon) prior to MS analysis.

#### Data acquisition

MS data was acquired using a Q-STAR Elite Q-TOF mass spectrometer (Applied Biosystems) with either a MALDI (matrix-assisted laser desorption ionization) or ESI (electron spray ionization) source depending on which source was installed at the time of sample submission for MS analysis.

#### MALDI-ionization

Samples were co-crystallized with 5 mg/ml α-cyano-4-hydroxy cinnamic acid (CHCA, Brüker Daltonics) matrix in 50% acetonitrile (ACN, Sigma), 0.1% trifluoro-acetic acid (TFA, Sigma) and spectra acquired with a 337 nm nitrogen laser operated at 20 Hz. Protein identification was based on peptide mass fingerprint (PMF) and sequencing data. The instrument was calibrated using Brüker's peptide calibration standard II using peaks at 757.39 and 2093.08 m/z. PMF spectra were acquired in positive ion mode using a range of 600-2500 m/z. MS/MS data was obtained by collision induced dissociation (CID), using argon as collision gas, from the 50 highest parent ions in the corresponding PMF spectra.

#### ESI-ionisation

Samples were loaded in Proxeon NanoES capillaries and ionized using IonSpray voltage of 900-1200 V. The instrument was calibrated using Glu-Fibrinopeptide B (Sigma-Aldrich) using fragment ions 246.15 and 1285.54 m/z. PMF spectra were acquired in positive ion mode using a range of 450 - 1500 m/z. MS/MS data obtained via Information Dependent Acquisition (IDA) method where doubly and triply charged parent ions were selected for fragmentation by collision induced dissociation (CID), using nitrogen as collision gas.

#### Data processing

Protein identification was performed by searching the NCBI database using the Mascot search engine. The following parameters were used for database searches with MALDI-QTOF PMF and sequencing data: monoisotopic mass, peptide charge +1, 50 ppm mass accuracy, trypsin as digesting enzyme with 1 missed cleavage allowed, carbamidomethylation of cysteine as a fixed modification, oxidation of methionine as allowable variable modification. For PMF data positive identifications equaled or exceeded the minimum significant score of 70. By MS/MS analysis, positive identifications required a minimum of two unique peptides, with at least one peptide having a significant ion score. Similar parameters were used to search ESI-QTOF PMF and sequencing data with only peptide charge set to +2 and +3. Exploratory analysis of the differentially expressed proteins was performed in MADIBA [34].

### Polyamine metabolite analysis

Synchronized *P. falciparum *3D7 cultures (two biological replicates) were treated with 2 mM cyclohexylamine as described for the transcriptome analyses. Parasites in mid-trophozoite (25 hpi) stages were selected for polyamine extraction. Triplicate samples of 20 ml parasite culture (~20% parasitemia) were harvested, pelleted by centrifugation at 2000 × g for 5 min (Boeco) and re-suspended in 4× equivalent pellet volumes of PBS. These wash steps were repeated four times in total. Subsequent cell counting was performed accurately in quadruplicate with a Neubauer Cell counting chamber (Weber).

PBS-washed cell pellets (1 ml) were subjected to protein precipitation using 5% (v/v) perchloric acid (PCA), followed by vigorous vortexing, incubation at 4°C (overnight) and subsequently precipitated by centrifugation at 16000 × g for 10 min at 4°C (Eppendorf). Polyamines were detected using benzoylation as previously reported with slight modification [50,51]. Five hundred μl of the 5% v/v PCA extract or 500 μl polyamine standard was added to glass tubes with pure 5% PCA as blank. An internal standard (1 nmol of 1,7-diaminoheptane (Sigma-Aldrich)) was included in the *P. falciparum *extracts to normalize for benzoylation efficiency. One ml 2.0 M NaOH was added to each reaction, vortexed after which 5 μl of benzoyl chloride (Sigma-Aldrich) was added. The reactions were incubated at 37°C for 30 min and stopped by adding 1 ml chloroform (<99%) to allow the separation of organic derivatized molecules. The tubes were centrifuged at 1500 × g (Medifuge) for 10 min, the aqueous phases were removed to a new glass tube containing additional 1 ml chloroform for a second separation step as above. The chloroform layers were pooled and evaporated under N_2 _gas. One ml 0.1 M NaOH was added to the white precipitate containing tubes and these were incubated overnight at room temperature. Subsequently, 1 ml chloroform was added to the aqueous phases, centrifuged at 1500 × g for 10 min and the chloroform phases evaporated under N_2_. Five hundred μl of 60% MeOH was used to redissolve benzoylated products. The methanol solutions were passed through 0.2 μm Minisart RC4 (Sartorius) HPLC-certified filters.

HPLC separation was performed using a WATERS System (Waters Corporation) with a 250 mm × 4.0 mm Luna C18(2) 5 μm reverse-phase (RP) column (Phenomenex). A Guard-Pak™ Precolumn steel housing (Waters Corporation) with μBondapak C18 HPLC pre-column inserts (Waters Corporation) was connected in-line. Isocratic solvent conditions were maintained using 60% MeOH:ddH_2_O at flow rate of 1 ml/min. All solvents were filtered using 0.2 μm cellulose acetate filters (Sartorius) in Millipore™ housing system (Millipore) and degassed prior to use. The HPLC pump system consisted of a WATERS 600 Controller (Waters Corporation) with a 600 Series WATERS Pump (Waters Corporation) and a WATERS 712 WISP Autosampler. UV absorbance was measured at 229 nm using a WATERS 996 Photodiode array (Waters Corporation). Peak integration was performed using the Empower 2 Software Edition (2006, Waters Corporation) with Apex™ Trac functionality.

## Authors' contributions

JvWB was the primary contributor to the gene expression experiments and subsequent data analysis, as well as its validation, and contributed to drafting the manuscript, LM was the primary contributor to the proteome investigation, BC was responsible for parasite drug treatments for morphological and gene expression experiments and performed RNA extractions; also contributed to optimizing cDNA labeling and subsequent hybridization and assisted in data analysis. SS carried out all mass spectrometry analysis for protein identification as well as subsequent data processing using MASCOT. ACvB helped with parasite cultures, data analysis and drafting of the manuscript. SR performed the metabolite analyses. AIL was involved with the final editing of the manuscript. LB was involved with the experimental planning, the metabolite analyses and drafting of the manuscript. DTM contributed to experimental planning, parasite drug treatments and drafting of the manuscript. All authors read and approved the final manuscript.

## Supplementary Material

Additional file 1 **Table S1. Pearson correlations between individual slides processed in the study**. Pearson correlations between technical and biological replicates of slides at each time point in solvent control and drug-treated samples. Click here for file

Additional file 2 **Table S2. Differentially expressed up- and downregulated genes at each time point following analysis**. PlasmoDB IDs and annotations of differentially expressed and unique up- and downregulated genes at each time point. Overall unique up- and downregulated genes are also listed. Click here for file

Additional file 3 **Table S3. Differentially affected proteins at each time point following cyclohexylamine inhibition**. Protein response (up- or downregulation), response time point and regulation of the encoding transcripts are indicated. Additionally, protein pI, MW, matched peptides and Protein Pilot scores are shown. Proteins involved in polyamine biosynthesis are indicated in bold. Click here for file
