# Supplementary material for: Plasmodium falciparum spermidine synthase inhibition results in unique perturbation-specific effects observed on transcript, protein and metabolite levels
Source: BMC Genomics. 2010 Apr 12;11:235. doi: 10.1186/1471-2164-11-235 (PMC2867828; doi:10.1186/1471-2164-11-235)
Supplement: Additional file 1 — Table S1. Pearson correlations between individual slides processed in the study. Pearson correlations between technical and biological replicates of slides at each time point in solvent control and drug-treated samples. [file 1471-2164-11-235-S1.DOC]

| **Slides** | **Pearson correlation (r)** |
| --- | --- |
| **Control 18h** |  |
| Technical replicates | 0.93 |
| Technical replicate 1 vs Biological replicate | 0.89 |
| Technical replicate 2 vs Biological replicate | 0.88 |
| **Control 25h** |  |
| Technical replicates | 0.86 |
| Technical replicate 1 vs Biological replicate | 0.67 |
| Technical replicate 2 vs Biological replicate | 0.64 |
| **Control 30h** |  |
| Technical replicates | 0.91 |
| Technical replicate 1 vs Biological replicate | 0.85 |
| Technical replicate 2 vs Biological replicate | 0.85 |
| **Cyclo 18h** |  |
| Technical replicates | 0.93 |
| Technical replicate 1 vs Biological replicate | 0.92 |
| Technical replicate 2 vs Biological replicate | 0.92 |
| **Cyclo 25h** |  |
| Technical replicates | 0.94 |
| Technical replicate 1 vs Biological replicate | 0.95 |
| Technical replicate 2 vs Biological replicate | 0.93 |
| **Cyclo 30h** |  |
| Technical replicates | 0.83 |
| Technical replicate 1 vs Biological replicate | 0.84 |
| Technical replicate 2 vs Biological replicate | 0.88 |
