# Supplementary material for: Plasmodium falciparum spermidine synthase inhibition results in unique perturbation-specific effects observed on transcript, protein and metabolite levels
Source: BMC Genomics. 2010 Apr 12;11:235. doi: 10.1186/1471-2164-11-235 (PMC2867828; doi:10.1186/1471-2164-11-235)
Supplement: Additional file 3 — Table S3. Differentially affected proteins at each time point following cyclohexylamine inhibition. Protein response (up- or downregulation), response time point and regulation of the encoding transcripts are indicated. Additionally, protein pI, MW, matched peptides and Protein Pilot scores are shown. Proteins involved in polyamine biosynthesis are indicated in bold. [file 1471-2164-11-235-S3.DOC]

| **SSP no. ^a^** | **Time point (hpi)** | **Protein ID**  **(PlasmoDB ID)** | **Protein** | | **Transcript (hpi)** | **Accession no. ^b^** | **pI** | **MW (kDa)** | **Matched peptides^c^** | **% Amino acid coverage^d^** | **ProteinPilot scores^e^** |
| --- | --- | --- | --- | --- | --- | --- | --- | --- | --- | --- | --- |
|  |  |  | **Response** | **Fold change** (relative to control) |  |  |  |  |  |  |  |
| 3302 | 18 | Hypothetical protein  (PF10_0325) | **↓** | 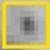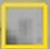  0.42 | **↓** (18) | Q8IJ74_PLAF7 | 5.62 | 32.84 | 5 | 37.2 | 8.06 |
| 0211 | 18 | Proteasome subunit alpha type 5, putative  (PF07_0112) | **↓** | 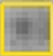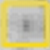  0.33 | **≈** | Q8IBI3_PLAF7 | 4.96 | 28.4 | 3 | 26.6 | 3.71 |
| 0402 | 18 | Endoplasmic reticulum-resident calcium binding protein  (PF11_0098) | **↓** | 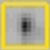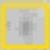  0.25 | **≈** | Q8IIR7_PLAF7 | 4.49 | 39.4 | 10 | 44.6 | 20.00 |
| 1609 | 18 | tubulin alpha-I chain  (PFI0180w) | **↓** | 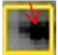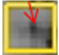  0.11 | **↓** (18, 25, 30) | TBA_PLAFK | 4.93 | 50.3 | 2 | 33.3 | 2.67 |
| 4603 | 18 | Phosphoglycerate hydratase (Enolase)  (PF10_0155) | **↓** | 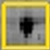*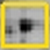*  0.34 | **≈** | ENO_PLAFA | 6.21 | 48.7 | 14 | 52.2 | 28.65 |
| **6604** | 18 | **Ornithine aminotransferase**  **(PFF0435w)** | **↓** | **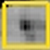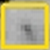**  **0.33** | **≈** | **OAT_PLAF7** | **6.47** | **46.1** | **5** | **45.7** | **10.11** |
| **6607** | 18 | **S-adenosylmethionine synthetase (Methionine adenosyltransferase)**  **(PFI1090w)** | **↓** | 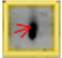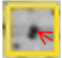  **0.35** | **≈** | **Q9GN14_PLAFA** | **6.28** | **44.8** | **11** | **41.5** | **21.75** |
| 7410 | 18 | Guanine nucleotide-binding protein, putative  (PF08_0019) | **↓** | 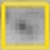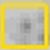  0.50 | **≈** | Q8IBA0_PLAF7 | 6.24 | 35.7 | 2 | 24.8 | 5.00 |
| 9401 | 18 | Glyceraldehyde-3-phosphate dehydrogenase  (PF14_0598) | **↓** | 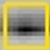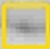  0.50 | **≈** | Q8T6B1_PLAFA | 7.59 | 36.6 | 10 | 58.8 | 20.30 |
| **4201** | 25 | **Uridine phosphorylase, putative**  **(PFE0660c)** | **↓** | 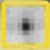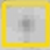  **0.45** | **↓** (18, 25, 30) | **Q8I3X4_PLAF7** | **6.07** | **26.9** | **12** | **54.3** | **15.93** |
| 0211 | 25 | Proteasome subunit alpha type 5, putative  (PF07_0112) | **↓** | 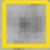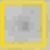  0.48 | **≈** | Q8IBI3_PLAF7 | 4.96 | 28.4 | 3 | 26.6 | 6.25 |
| 0402 | 25 | Endoplasmic reticulum-resident calcium binding protein  (PF11_0098 ) | **↓** | 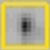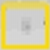  0.22 | **≈** | Q8IIR7_PLAF7 | 4.49 | 39.4 | 8 | 40.2 | 16.95 |
| **6607** | 25 | **S-adenosylmethionine synthetase**  **(PFI1090w)** | **↓** | 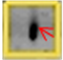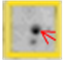  **0.45** | **≈** | **Q9GN14_PLAFA** | **6.28** | **44.8** | **11** | **41.5** | **19.75** |
| 2905 | 25 | Heat shock protein  (PFI0875w) | **↓** | 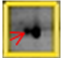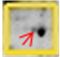  0.23 | **≈** | Q8I2X4_PLAF7 | 5.18 | 72.4 | 26 | 44.9 | 54.89 |
| 1609 | 25 | tubulin alpha-I chain  (PFI0180w) | **↓** | 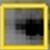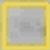  0.19 | **↓** (18, 25, 30) | TBA_PLAFK | 4.93 | 50.3 | 2 | 33.3 | 4.11 |
| 9401 | 30 | Glyceraldehyde-3-phosphate dehydrogenase  (PF14_0598) | **↓** | 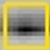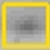  0.39 | **≈** | Q8T6B1_PLAFA | 7.59 | 36.6 | 10 | 58.8 | 14.33 |
| 2501 | 30 | **Adenosine deaminase**  **(PF10_0289)** | **↓** | 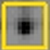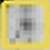  **0.33** | **↓** (18, 25, 30) | **Q8IJA9_PLAF7** | **5.41** | **42.5** | **3** | **31.1** | **11** |
| 1407 | 30 | Ran binding protein 1  (PFD0950w) | **↓** | 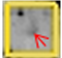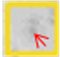  0.40 | **≈** | Q76NN6_PLAF7 | 4.92 | 33.2 | 4 | 28.2 | 7.00 |
| 1609 | 30 | tubulin alpha-I chain  (PFI0180w) | **↓** | 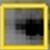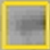  0.31 | **↓** (18, 25, 30) | TBA_PLAFK | 4.93 | 50.3 | 2 | 33.3 | 5.44 |
| 2905 | 30 | Heat shock protein  (PFI0875w) | **↓** | 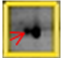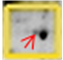  0.22 | **≈** | Q8I2X4_PLAF7 | 5.18 | 72.4 | 22 | 41.1 | 42.81 |
| 6703 | 30 | Putative pyruvate kinase  (PFF1300w) | **↑** | 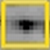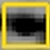  2.19 | **↓** (25) | Q6LF06_PLAF7 | 7.50 | 55.7 | 3 | 19.0 | 6.70 |

a= Standard Spots (SSP) number assigned to each unique 2D protein-gel spot detected across matched gels; used to create the MatchSet master gel against which all spots are compared to establish statistical significance

b= UniProtKB/TrEMBL protein accession number assigned by MASCOT in ProteinPilot

c=Number (N) of short peptide sequences successfully identified within the predicted protein sequence (confidence level ≥95%)

d=Percentage of residues mapped against total protein sequence (confidence level ≥95%)

e= A measure of the total amount of evidence for a protein detected by ProteinPilot, calculated using all of the peptides detected for the protein. Note: the score does not indicate the percent confidence for the identification of a protein

**↓**=downregulated

**↑**=upregulated

**≈**=unchanged

Boxed images depict protein spots estimated to be significantly differentially expressed between 2D protein-gel profiles of untreated (left panel) and treated (right panel) parasites
